# 

**Published:** 1882-09

**Authors:** 


					﻿SEPTEMBER, 1882.
TWENTY-NINTH YEAR.
HALL’S
JOURNAL
OF
HEALTH
E. H. GIBBS, A.M., M.D., Editor,
No. 135 EIGHTH STREET (Near Broadway), NEW YORK.
TERMS: $2 a Year, or $1 for Six Months. Single number, 20 cts. <
				

